# da Vinci robot-assisted keyhole neurosurgery: a cadaver study on feasibility and safety

**DOI:** 10.1007/s10143-014-0602-2

**Published:** 2014-12-18

**Authors:** Hani J. Marcus, Archie Hughes-Hallett, Thomas P. Cundy, Guang-Zhong Yang, Ara Darzi, Dipankar Nandi

**Affiliations:** 1 The Hamlyn Centre for Robotic Surgery, Institute of Global Health Innovation, Imperial College London, Paterson Building (Level 3), Praed Street, London, W2 1NY UK; 2 Department of Neurosurgery, Imperial College Healthcare NHS Trust, London, UK

**Keywords:** Image guided intervention, Minimally invasive surgery, Neurosurgery, Robotic surgery

## Abstract

The goal of this cadaver study was to evaluate the feasibility and safety of da Vinci robot-assisted keyhole neurosurgery. Several keyhole craniotomies were fashioned including supraorbital subfrontal, retrosigmoid and supracerebellar infratentorial. In each case, a simple durotomy was performed, and the flap was retracted. The da Vinci surgical system was then used to perform arachnoid dissection towards the deep-seated intracranial cisterns. It was not possible to simultaneously pass the 12-mm endoscope and instruments through the keyhole craniotomy in any of the approaches performed, limiting visualization. The articulated instruments provided greater dexterity than existing tools, but the instrument arms could not be placed in parallel through the keyhole craniotomy and, therefore, could not be advanced to the deep cisterns without significant clashing. The da Vinci console offered considerable ergonomic advantages over the existing operating room arrangement, allowing the operating surgeon to remain non-sterile and seated comfortably throughout the procedure. However, the lack of haptic feedback was a notable limitation. In conclusion, while robotic platforms have the potential to greatly enhance the performance of transcranial approaches, there is strong justification for research into next-generation robots, better suited to keyhole neurosurgery.

## Introduction

**Fig. 1:**
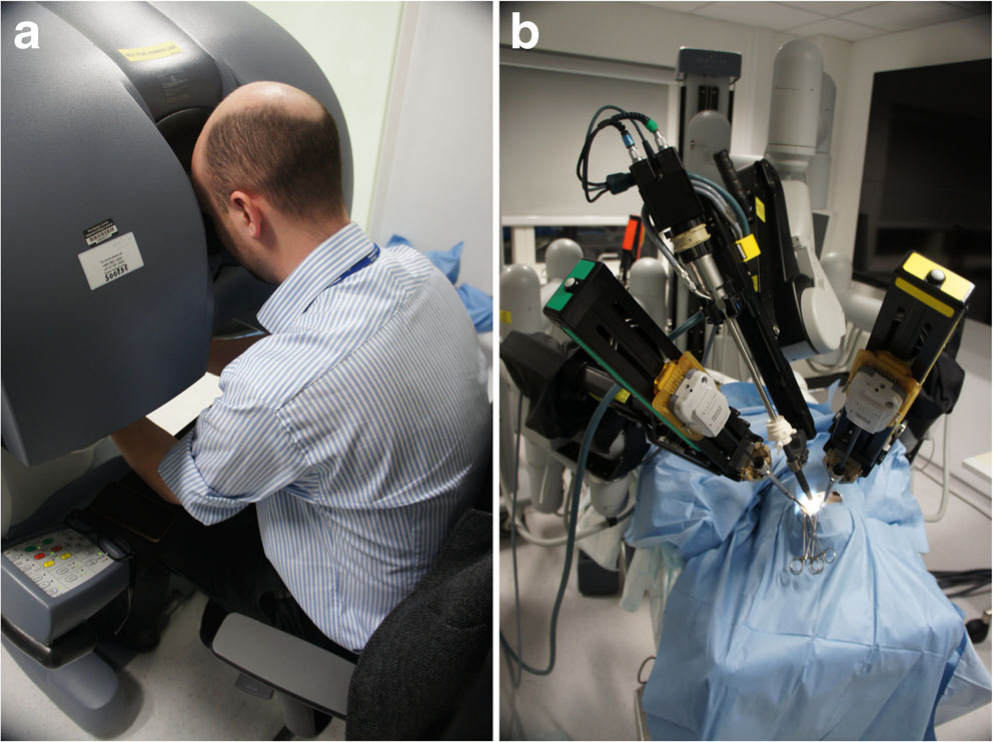
Arrangement of the da Vinci master-slave system. **a** The surgeon is seated comfortably at the console and remotely controls the robots’ actions. **b** The surgical cart includes an endoscope and instrument arms and carries out the procedure. Note the difficulty in parallel insertion of the instrument arms through a single keyhole craniotomy

Robotic platforms that further enhance surgical skills have the potential to improve the safety and efficacy of keyhole neurosurgery [9, 11, 22]. At present, the most widely used surgical robot worldwide is the da Vinci system (Intuitive Surgical, CA, USA), a master-slave system designed for minimally invasive surgery (MIS) in which the surgeon remotely controls the robots’ actions (Fig. 1). The camera arm includes two lenses, providing surgeons with a high-resolution stereoscopic image of the operative field. The instrument arms contain articulated endo-wrists, which increase surgical dexterity. In addition, the system allows for motion scaling and tremor filtering. To date, the da Vinci system has been used in a broad range of procedures [2, 12] and has achieved substantial clinical penetration in fields such as urology [5, 13].

Recently, a group has reported the application of the da Vinci surgical system to assist with the supraorbital subfrontal approach through an eyebrow skin incision in a cadaver study [6]. While the authors concluded that such robot-assisted approaches were probably feasible, they noted several drawbacks including the lack of suitable instruments such as bone cutters and the risk of arm collisions and highlighted the need for further studies. The goal of the present cadaver study was therefore to confirm and extend these preliminary findings by applying the da Vinci surgical system to a range of keyhole neurosurgical approaches.

## Methods

Ethical approval was obtained from the Proportionate Review Sub-Committee of the National Research Ethics Service (NRES) Committee East Midlands. A formalin-fixed cadaver head was obtained from the Department of Anatomy, Imperial College London. Surgical residents (HJM and AHH) performed the procedures under the supervision of the senior clinical authors (DN and AD), who are experienced in minimally invasive neurosurgery and robot-assisted surgery, respectively.

The cadaver head was secured in a Mayfield clamp and a Budde Halo retractor system attached (Integra, NJ, USA). An Albert Wetzlar operating microscope (Albert Wetzlar GmbH, Wetzlar, Germany) and high-speed drill (B. Braun, Melsungen, Germany) were used to fashion several previously described keyhole craniotomies, approximately 20–30 mm in diameter [15, 17]: supraorbital subfrontal, retrosigmoid and supracerebellar infratentorial. Craniotomies were not extended to accommodate the da Vinci system; instead, the aim was to determine the suitability of the robot to typical keyhole craniotomies. In each approach, a simple durotomy was performed, and the flap was retracted.

The standard da Vinci robotic system was used intradurally, and arachnoid dissection was performed towards the deep-seated cisterns. The surgeon remained non-sterile at the robot console, while an assistant was available throughout the procedure to exchange robotic instruments. Both 0° and 30° upwards facing 12-mm endoscopes were introduced into the keyhole craniotomy for visualization. Standard 8-mm and smaller 5-mm instruments were used for tissue manipulation. Throughout each procedure, a detailed feedback was obtained, including images of the operating room arrangement and of the endoscope feed.

## Results

**Fig. 2:**
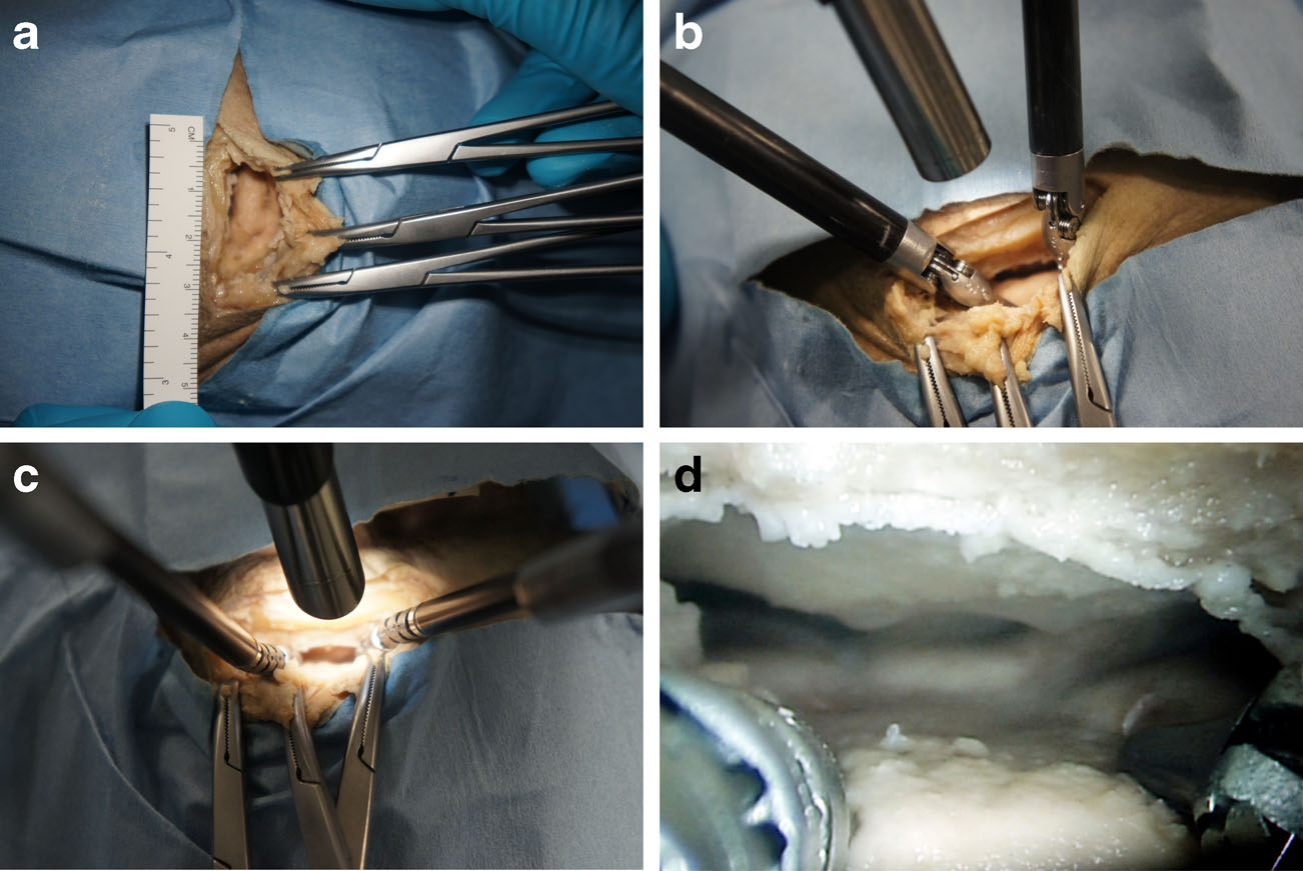
Left supraorbital subfrontal approach through an eyebrow incision demonstrating the following: **a** keyhole craniotomy approximately 25 × 15 mm in size, **b** a 12-mm endoscope and two standard 8-mm instruments were unable to enter the keyhole simultaneously, **c** a 12-mm endoscope and two smaller 5-mm instruments were also unable to enter the keyhole simultaneously, and **d** endoscopic visualization was therefore limited

It was not possible to simultaneously pass the 12-mm endoscope and instruments through the keyhole craniotomy in any of the approaches performed, irrespective of whether two standard 8-mm instruments or smaller 5-mm instruments were used (Fig. 2). Instead, the endoscope had to be placed outside the craniotomy, limiting the illumination, magnification, and wide-angle view provided.

**Fig. 3:**
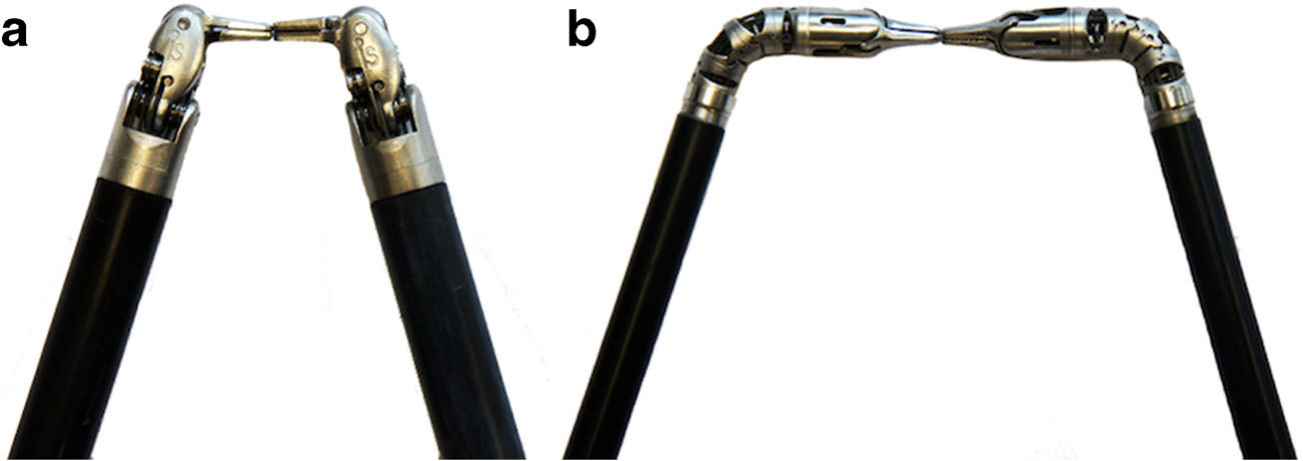
Comparison of **a** 8-mm da Vinci instruments with articulated wrist joints and (**b**) 5-mm da Vinci instruments with tentacle-like continuum tool shafts

Dissection with the da Vinci instruments was restricted to superficial structures, approximately 20 mm from the craniotomy. The large instrument arms could not be placed in parallel through the keyhole craniotomy and, therefore, could not be advanced to the deep cisterns without significant clashing (Fig. 1). The smaller 5-mm instruments were comparatively easier to pass through the keyhole craniotomy but utilized tentacle-like continuum tool shafts rather than the articulated wrist joints that characterize standard 8-mm instruments. The result, paradoxically, was that the smaller 5-mm instruments had less dexterity than the standard 8-mm instruments in the spatially constrained intracranial cisterns (Fig. 3). The range of 5-mm instruments was also limited, with no bipolar forceps or suction-irrigation available. Nonetheless, all the robotic instruments used during the study allowed for greater dexterity than existing rigid tube shaft instruments.

The da Vinci console offered considerable ergonomic advantages over the existing operating room arrangement, allowing the operating surgeon to remain non-sterile and seated comfortably throughout the procedure (Fig. 2). The visualization provided by the 3-D endoscope provided an immersive view of the operative field. Control of the instruments was intuitive and allowed for motion scaling and tremor filtering. However, the lack of haptic feedback was a notable limitation.

## Discussion

In this cadaver study, it has been demonstrated that use of the standard da Vinci robotic system in keyhole transcranial endoscope-assisted microsurgery is neither safe nor feasible. Arguably, the greatest role of surgical robots is as a “great leveler”, allowing surgeons to perform keyhole approaches when they would otherwise resort to open surgery [10]. To this end, technically challenging keyhole neurosurgical approaches are ideal targets for such surgical robots. Regrettably, in its present form, the da Vinci robotic platform is ill-suited to brain surgery given its multiple large and bulky arms, limited selection of instruments and lack of haptic feedback.

In a related study, Thakre et al. sought to evaluate the performance of the da Vinci platform in increasingly small workspaces [18]. It was noted that a cube had to be at least 40 mm in size to simultaneously pass both the endoscope and instruments, at least 50 mm in size to perform standard surgical drills and at least 60 mm in size to do so without significant collision between the instrument arms (albeit with difficulty). These findings broadly corroborate those of the present study, in which it was not possible to simultaneously pass both the endoscope and instruments through keyhole craniotomies approximately 20–30 mm, and instrument clashes prevented dissection towards the deep intracranial cisterns (see Table 1).

**Table 1:** Summary of the limitations of the da Vinci platform in small working spaces using data from the present cadaver study (size <40 mm) and the previous preclinical study by Thakre et al. (size ≥40 mm)

	Size (mm)	Visualization with endoscope	Manipulation with 8-mm instruments	Immersive console
Keyhole craniotomy	20	Limited	Limited	Full
	30	Limited	Limited	Full
	40	Full	Limited	Full
	50	Full	Full but with instrument collisions	Full
	60	Full	Full without collisions, but with difficulty	Full
Minicraniotomy	70 or greater	Full	Full	Full

Previous preclinical study by Thakre et al. [18]

The present cadaver study has several inherent limitations. The cadaver brain was formalin-fixed and was therefore not as a compliant as the living brain, though pathology resulting in cerebral oedema can be similarly tense. The cadaver brain also did not allow for assessment of haemostasis, which is frequently cited as a limitation of keyhole approaches. Animal studies might better address the issue of haemostasis, but there are very few animal models with brains of a similar size to humans; such studies would be logistically difficult to organize and also raise ethical concerns.

While several studies have reported the use of the da Vinci robot in spinal and peripheral nerve surgery [1, 4, 7, 14, 16, 19–21], only one previous cadaver study has described the use of the da Vinci robot in keyhole brain surgery [6]. Hong et al. described the application of the da Vinci robot to the keyhole supraorbital approach and, in contrast to the present study, found that it was generally feasible, though they did comment on instrument clashes and lack of proper tools. We speculate that this discrepancy may be due to their longer incision and larger craniotomy and their use of brain retractors.

Recently, a group from the University of Washington has proposed new multiport approaches to the anterior cranial fossa that are better suited to the da Vinci robotic platform [3]. In order to overcome the narrow funnel effect generated from arms in close proximity, and the steep angle of approach to the skull base, they suggest transnasal and bilateral medial orbital ports for the camera and instruments, respectively. While such novel approaches may be more viable from a technical standpoint, they carry a greater risk of approach-related morbidity than standard keyhole approaches.

Alongside the technical challenges to the use of the da Vinci robotic platform in keyhole neurosurgery highlighted in the present study, several other barriers also exist [8]. Arguably, the greatest drawback to existing surgical robots is economic. The current da Vinci robot, for example, is priced at over $2 m, carries substantial maintenance costs and requires additional training of the surgeons and nurses involved with its use. Next-generation robotic platforms may mitigate these limitations. Over time, the large and expensive multipurpose robots of today are likely to be replaced by smaller and more affordable robots tailored to particular procedures [8].

## Conclusions

Keyhole transcranial endoscope-assisted microsurgical techniques are technically challenging approaches that may greatly benefit from surgical robotics. However, the most widely used surgical robot worldwide today, the da Vinci platform, is neither safe nor feasible to use in keyhole neurosurgery. There is therefore strong justification for research into next-generation robots, better suited to such approaches.
